# EFFECTS OF MULTICOMPONENT EXERCISE INTERVENTION ON SARCOPENIA PREVENTION IN COMMUNITY-DWELLING OLDER ADULTS

**DOI:** 10.1093/geroni/igae098.2819

**Published:** 2024-12-31

**Authors:** Kewei Shi, Lingxiao He, Ya Fang

**Affiliations:** Xiamen University, Xiamen, Fujian, China (People’s Republic); Center for Health Economics, Policy and Aging Research, Xiamen city, Fujian, China; Xiamen University, Xiamen, Fujian, China (People’s Republic)

## Abstract

**Objective:**

To assess the effects of multicomponent exercise intervention on sarcopenia and its components among community-dwelling older adults. Design, Setting, and Participants: In a randomized controlled trial conducted in 11 communities across Xiamen, China, with 200 older adults (aged > 65 years) being randomly assigned to an exercise group (n=100) or a control group (n=100).

**Interventions:**

The exercise group followed a multicomponent exercise program, consisting of resistance, balance and flexibility training, twice a week for six months. The control group received monthly sarcopenia-related health education. Outcome measures: The primary outcome was the incidence of sarcopenia. The secondary outcomes included muscle mass, muscle strength (knee extension and grip strength), the Short Physical Performance Battery (SPPB) score, 4-meter gait speed, and 5-time chair stand test (CST) performance. Generalized mixed-effects model was performed for primary outcome comparison between two groups. Repeated measures ANCOVA was performed to evaluate the effects on secondary outcomes.

**Results:**

The exercise group showed a decrease of 4.3% on the incidence of sarcopenia after 6-month intervention, with no significant difference from the control group (P=0.171). The exercise group demonstrated significant improvements in knee extension (β=1.95, 95% CI [0.082, 3.84]), grip strength (β=2.07, 95% CI [0.857, 3.290]), SPPB score (β=1.99, 95% CI [1.600, 2.380]), gait speed (β=0.182, 95% CI [0.128, 0.235]) and CST (β=-1.590, 95% CI [-2.400, -0.786]). No between-group difference was observed in muscle mass (P=0.767).

**Conclusion:**

A 6-month multicomponent training can significantly improve physical function and muscle strength but not muscle mass among community-dwelling older adults.

